# p53 at the crossroad of DNA replication and ribosome biogenesis stress pathways

**DOI:** 10.1038/s41418-022-00999-w

**Published:** 2022-04-20

**Authors:** Mikael S. Lindström, Jiri Bartek, Apolinar Maya-Mendoza

**Affiliations:** 1 Karolinska Institutet, Department of Medical Biochemistry and Biophysics, Division of Genome Biology, Science for Life Laboratory, Stockholm, Sweden; 2 Danish Cancer Society Research Center, Genome Integrity Group, Copenhagen, Denmark; 3 Danish Cancer Society Research Center, DNA Replication and Cancer Group, Copenhagen, Denmark

**Keywords:** Cancer, Cell biology

## Abstract

Despite several decades of intense research focused on understanding function(s) and disease-associated malfunction of p53, there is no sign of any “mid-life crisis” in this rapidly advancing area of biomedicine. Firmly established as the hub of cellular stress responses and tumor suppressor targeted in most malignancies, p53’s many talents continue to surprise us, providing not only fresh insights into cell and organismal biology, but also new avenues to cancer treatment. Among the most fruitful lines of p53 research in recent years have been the discoveries revealing the multifaceted roles of p53-centered pathways in the fundamental processes of DNA replication and ribosome biogenesis (RiBi), along with cellular responses to replication and RiBi stresses, two intertwined areas of cell (patho)physiology that we discuss in this review. Here, we first provide concise introductory notes on the canonical roles of p53, the key interacting proteins, downstream targets and post-translational modifications involved in p53 regulation. We then highlight the emerging involvement of p53 as a key component of the DNA replication Fork Speed Regulatory Network and the mechanistic links of p53 with cellular checkpoint responses to replication stress (RS), the driving force of cancer-associated genomic instability. Next, the tantalizing, yet still rather foggy functional crosstalk between replication and RiBi (nucleolar) stresses is considered, followed by the more defined involvement of p53-mediated monitoring of the multistep process of RiBi, including the latest updates on the RPL5/RPL11/5 S rRNA-MDM2-p53-mediated Impaired Ribosome Biogenesis Checkpoint (IRBC) pathway and its involvement in tumorigenesis. The diverse defects of RiBi and IRBC that predispose and/or contribute to severe human pathologies including developmental syndromes and cancer are then outlined, along with examples of promising small-molecule-based strategies to therapeutically target the RS- and particularly RiBi- stress-tolerance mechanisms to which cancer cells are addicted due to their aberrant DNA replication, repair, and proteo-synthesis demands.

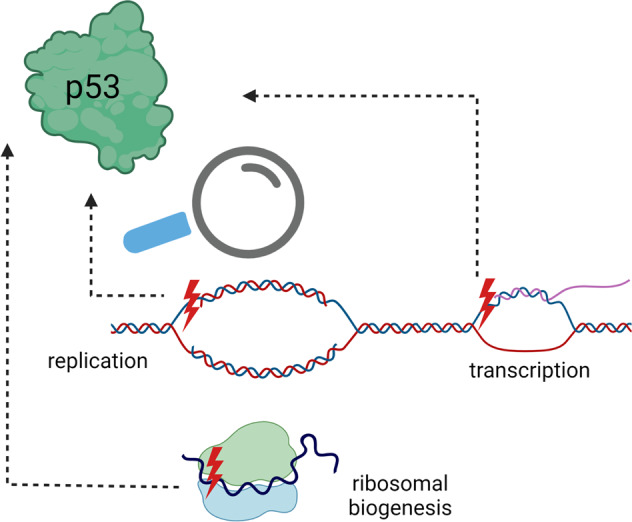

## Facts

1. p53 plays fundamental, yet mechanistically not entirely understood roles in the regulation of genome replication and ribosome biogenesis.
2. By regulating the speed of DNA replication fork progression and cellular responses to replication stress, p53 guards against genomic instability.
3. Defects in ribosome biogenesis activate a RPL5/RPL11/5 S rRNA-MDM2-p53-mediated cell cycle checkpoint and G1 phase arrest.
4. Replication stress and aberrant ribosome biogenesis fuel tumorigenesis and favor the selection of p53 mutations, while unmasking actionable cancer vulnerabilities.

## Open questions

1. What is the precise molecular basis of the tantalizing p53-centered functional crosstalk between DNA replication and ribosome biogenesis?
2. How and why did p53 activity become so intimately linked to DNA replication and ribosome biogenesis?
3. Do any of the cancer-associated gain-of-function p53 mutant proteins impact DNA replication and/or RiBi, and if yes through which mechanism?
4. Which small-molecule modulators of RS- and RiBi stress-tolerance pathways are best suited for cancer treatment and in which tumor (sub)types?

## Introduction

The year 2022 marks the 30th anniversary of one of the milestones in cell biology, discoveries that inspired the concept of p53 as the guardian of the genome [1]. A year later, p53 was selected for the molecule of the year award [2]. These events, without doubt, paved the way for a better understanding of some fundamental aspects of cell biology and pathology. Next to insulin, p53 is the most studied protein in science history, at least in part due to p53’s many talents and the fact that it is commonly altered in cancer [3]. Today, it is well established that mutations in *TP53* are shared by most types of human tumors [4]. Biologically, p53 is at the heart of responses to numerous cellular stress insults, with DNA damage being the first stressor shown to stabilize p53 [5]. Under physiological conditions, the level of p53 is maintained low mainly by the E3 ubiquitin ligase MDM2 that targets p53 for degradation [6]. *MDM2* contains a p53 DNA-binding site, therefore, its expression can be regulated by p53’s abundance and transcriptional activity. As part of this intricate interplay, MDM2 can bind p53, potentially inhibiting *MDM2* transactivation. In this regulatory feedback loop, p53 regulates *MDM2* at the transcription level and MDM2 regulates the activity of p53 [7, 8]. The p53-MDM2 regulatory loop generates oscillations at the level of both proteins, in response to the intensity and duration of diverse stressors. Such oscillations may help cells to recover from DNA damage and avoid excessive cell death or senescence due to chronic p53 activation [9–11]. The transcriptional function of p53 is initiated by its direct binding to DNA [12, 13]. Early reports highlighted p53 as an important regulator of cell cycle progression through controlling expression of the CDK inhibitor p21, particularly in response to genotoxic insults [14]. Upon severe DNA damage, p53 regulates expression of genes whose products are involved in cell death mechanisms, such as down-regulation of *BCL-2* [15] and up-regulation of *NOXA* and *PUMA* [16].

The exquisite regulation of p53 comes in many flavors, such as post-translational modifications, which influence p53 stability or specificity of its target genes. Phosphorylation [17] can lead to stabilization and nuclear accumulation of p53 [18]. Several kinases can phosphorylate p53 at different sites and with some level of redundancy [19]. For instance, the DNA damage-activated protein kinase (DNA-PK) phosphorylates p53 at Ser15 and Ser37, the former resulting in the dissociation of the p53-MDM2 complex [20].

Genotoxic stressors activate the multifaceted cellular signaling network called the DNA damage response (DDR). In response to oncogene-induced DNA damage, the DDR including p53 provides a biological barrier against tumor progression [21, 22]. DDR senses the damage and, depending on the severity of the insult, induces cell cycle delay and DNA repair, senescence, or cell death. Together with p53, two phosphoinositide-3-kinase-related protein kinases, ATM and ATR, are key DDR components. Both ATM and ATR can phosphorylate p53, thereby contributing to the DNA damage-induced G1/S and G2/M checkpoints [23]. The checkpoint-induced p53 transcriptional activation requires ATM and CHK2-dependent phosphorylation at S15 and S20, respectively [24, 25]. p53 transactivates p21, inducing its accumulation. p21 binds and inhibits the cyclin E/CDK2 and cyclin A/CDK2 kinase complexes and inhibits them [26], leading to G1/S cell cycle arrest, preventing DNA synthesis.

p53 is also regulated by acetylation mediated by the acetyltransferases p300, PCAF, and CBP. Acetylation might not be critical for p53 activation, as unacetylated p53 retains its ability to induce the p53-MDM2 feedback loop, nevertheless, the loss of p53 acetylation might impact the p21-mediated stress response [27]. MDM2 promotes p53 deacetylation by recruiting a complex containing the histone deacetylase 1 (HDAC1). The HDAC complex binds MDM2 in a p53-independent manner and deacetylates p53. Interestingly, acetylated p53 lysine residues overlap with the residues that can be ubiquitylated, therefore, the acetylation of p53 promotes its stability by preventing the MDM2-dependent ubiquitylation, while HDAC1 activity promotes the degradation of p53 by removing its acetylation [28]. Furthermore, several other deacetylases regulate p53 function. Thus, HDAC 1, 2, and 3 attenuate p53 function, specifically, the binding of p53 to the BAX promoter was reduced in the presence of HDACs [29]. Other post-translational modifications of the p53 lysine residues include mono and poly-ubiquitylation, sumoylation, neddylation, and methylation [30]. Lysine methylation depends on DNA damage and regulates subsequent acetylation events [31, 32]. Whereas sumoylation may promote p53 transcriptional activity [33] and/or its retention in the cytoplasm [34], neddylation of p53 appears to inhibit p53-mediated transcriptional activation [35]. Other, less well-characterized regulatory modifications of p53 include methionine oxidation, the addition of O-linked N-acetylglucosamine, prolyl-isomerization, and NAD-dependent ADP-ribosylation [36]. The potential code dictated by post-translational modifications of p53 suggests a very complex regulation of its cellular function(s), and whether these modifications are functionally redundant or unique remains to be investigated.

To add an extra layer of complexity, at least nine different isoforms of p53 can be expressed from its gene *TP53* in human cells [37]. Two additional genes, *TP63* and *TP73* encoding p63 and p73, respectively, share some degree of amino acid sequence identity with the transactivation domain, the DNA-binding domain, and the oligomerization domain of p53. Currently, p53, p63, and p73 constitute the p53 family of transcription factors [38], whereby p63 and/or p73 have some redundant functions to p53. Indeed, p73 can activate some p53-target genes, arrest the cell cycle, and regulate apoptosis [39, 40]. In contrast to p53, p63 is essential for ectodermal differentiation, while the lack of p53 does not impair development in murine models [41]. Therefore, the p53 family members regulate several fundamental biological processes, spanning from development (p63 and p73) to cell cycle control upon DNA damage (p53, p63, and p73) [42]. p53 knock-out mice develop normally, however, the animals are tumor prone by the age of 6 months [43]. In humans, p53 function and regulation might be more complicated than in mice, with no reports of p53-null children born, and the human p53-null embryos being most likely nonviable [44, 45]. Therefore, the role of p53 in human early development differs from that in mice, particularly in terms of genome maintenance. Thus, in human embryonic stem cells (hESC) p53 is unable to transactivate its target genes upon stress [46] and therefore cells may accumulate genomic instability after multiple divisions [47]. In mouse embryonic stem cells (mESCs) the situation is different, since p53 can transactivate its target genes efficiently, resulting in a relatively low level of mutations due to their more robust repair capacity and/or elimination of genomically unstable cells by p53-induced apoptosis [48]. Furthermore, p53 might promote lineage commitment, as *TP53−/−* hESCs fail to differentiate into neural progenitor cells [49]. In any case, the role of p53 in regulating cell death during the organismal lifetime is crucial, keeping the balance between cell proliferation, DNA repair, and genome stability.

It is probable that all stressors impacting cell function lead to post-translational modification(s) and activation of p53. The list of cellular stressors includes, but is not limited to, oncogene activation, DNA damage, telomere shortening, replication stress, dysregulated transcription, altered ribosome biogenesis, hypoxia, nutrient deprivation, mitochondrial stress, mitotic defects, thermal shock, protein misfolding, and ROS accumulation, with more likely to be identified [50]. To limit overlap with many excellent reviews about p53 published over the years, here we will mainly discuss the recent discoveries and open issues related to p53 involvement in response to ribosome biogenesis and DNA replication stresses, a busy crossroad in cell homeostasis that is currently in the spotlight of biomedical research [51].

## p53 and replication stress

**Fig. 1:**
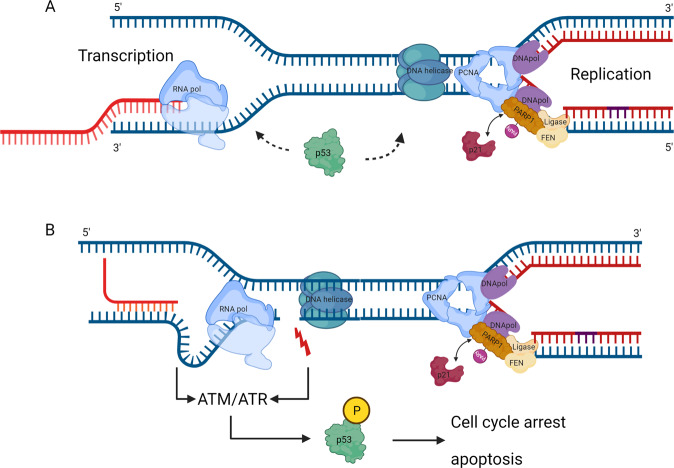
p53 helps cells to avoid transcription-replication conflicts and repair their consequences. **A** Under normal S phase p53 prevents DNA topological stress that could occur through conflicts between the transcription and replication machineries [55]. **B** Deregulated transcription may induce topological barriers that interfere with DNA replication resulting in replication stress (RS). DNA breaks can also induce RS. In response to such stress, p53 is activated by ATM/ATR-regulated signaling pathways, resulting in repair, cell cycle arrest or apoptosis.

Any condition that negatively impacts DNA synthesis and compromises replication fork integrity qualifies as replication stress (RS). RS typically causes fork arrest and collapse, however, it can also accelerate the speed of fork progression, activating the DNA damage response [52–54]. Accumulating evidence indicates that p53 regulates genomic DNA replication under both normal circumstances and RS [55]. p53 associates with active replication forks and is central in response to RS. When forks stall, p53 recruits repair proteins to facilitate fork re-start [56]. Simultaneously, stalled forks trigger signaling kinases that modify and stabilize p53 (Fig. 1A, B). Wild-type p53 stabilized during such S-phase response is, however, unable to regulate transcription of target genes [57]. As p53-null or -mutant cells lack the long-established p53-p21 G1-checkpoint, they enter and progress through S phase regardless of the presence of DNA damage. Furthermore, DNA breaks observed in cells lacking the G1/S checkpoint are caused by RS, consistent with slow replication speed and reduced origin firing [58].

The ATR/CHK1 signaling pathway plays a major role in the response to RS [59]. Perturbations in the replication machinery cause accumulation of single-stranded DNA that, in turn, recruits the replication protein A (RPA). RPA located at stalled forks is recognized by ATR in complex with ATRIP, ETAA1 and the complex RAD9-RAD1-HUS1 (9-1-1). The 9-1-1 complex recruits TopBP1, which together with ETAA1 contributes to ATR activation [60]. Activated ATR phosphorylates multiple substrates including CHK1 at Ser317 and Ser345. Phosphorylated CHK1 can further phosphorylate other proteins, such as CDC25A and TRESLIN, allowing the intra-S phase regulation of origin firing and cell cycle arrest upon RS. Other ATR substrates include MCMs, SMARCAL, WRN, and p53 [61], the phosphorylation of which by ATR reflects the extent and type of DNA damage [62–64]. The interaction between TRESLIN-MTBP and TopBP1 plays a crucial role in the firing of DNA replication origins under normal conditions; this interaction is regulated by CDK activity to ensure the proper replication program including its timing [65, 66]. While p53 helps to mitigate RS, whether such a role involves direct or indirect p53 signaling is still under investigation. Some p53 mutants (mutp53s) aberrantly reduce the ATR-mediated response to RS through binding to TopBP1, thereby impairing ATR activation. Mutp53s can also override the CDK2 regulation and promote origin firing by facilitating the interaction between TopBP1 and TRESLIN [67].

p53 avoids RS at the telomeres, specifically, telomeres contain difficult-to-replicate repetitive DNA sequences protected by capping proteins [68] and RS at telomeres can induce p53-mediated premature senescence [69]. The ATM/ATR-p53-p21 pathway monitors telomere capping after DNA replication and delays mitotic entry in the presence of uncapped telomeres which resemble unrepaired double-stranded DNA breaks [70]. Furthermore, uncapped telomere-activated ATM-CHK2/ATR-CHK1 signaling triggers CDC25C degradation, thereby preventing mitotic progression [71]. These examples illustrate the complexity and plasticity of cellular responses to RS.

Another example of p53’s versatility in terms of dealing with RS is p53’s role in silencing the Long Interspersed Element 1 (LINE-1). LINE-1 is a family of autonomous retrotransposons that are active in the human genome. LINE-1 contains two open reading frames (ORF1p and ORFp2) that are necessary for this element to spread to new genomic loci. Under physiological conditions, LINE-1 is silenced in somatic cells but cases of derepression and overexpression have been observed in cancer [72]. LINE-1 expression induces replication stress and activates the DDR [73, 74]. p53 may protect cells from LINE-1-induced RS by triggering G1 arrest, furthermore, p53 stimulates local deposition of repressive histone markers at the transposons, thereby restricting the autonomous copies of these potentially harmful mobile elements [75].

## p53 and the fork speed regulatory network

Our previous work showed that p53 depletion caused reduced fork speed and accumulation of arrested forks. Interestingly, fork defects were alleviated by concomitant double-knockdown of p53 and PARP1 [52]. Upon DNA damage and during early apoptosis, PARP1 adds PARy residues into p53, a modification that inhibits the binding of p53 to its consensus DNA sequence [76, 77]. Moreover, p53 can also bind PARy residues, which controls p53-DNA interaction [78]. Indeed, PARylation of p53 may impact the p53 interactome, transcription, and replication [79], at least in part by preventing the CRM1-mediated nuclear export of p53 [80].

Sensing chromosomal breaks and rearrangements emerging from defective forks or unfinished DNA replication is another major role of p53, with such checkpoint potentially operating directly at the fork level and/or transcriptionally regulating factors involved in preserving fork integrity. Notably, chronic induction of p21 in a p53-independent manner, mimicking p21 expression triggered by deregulated cytokines, hormones, or growth factors in advanced p53-mutant cancers, resulted in RS and genomic instability, reflecting the inability of PCNA to interact with and regulate the degradation of the replication licensing factors CDT1 and CDC6 [81]. p53, CtBP, and PARP1 form a co-repressor complex required for *p21* gene repression. Upon DNA damage, PARylated PARP1 gets released from this complex, allowing recruitment of a co-activator p53/p300 and hence *p21* transcription [82]. Together with PARP inhibitors preventing the up-regulation of p21 [52], the above evidence suggests a molecular pathway that regulates genomic DNA synthesis.

**Fig. 2:**
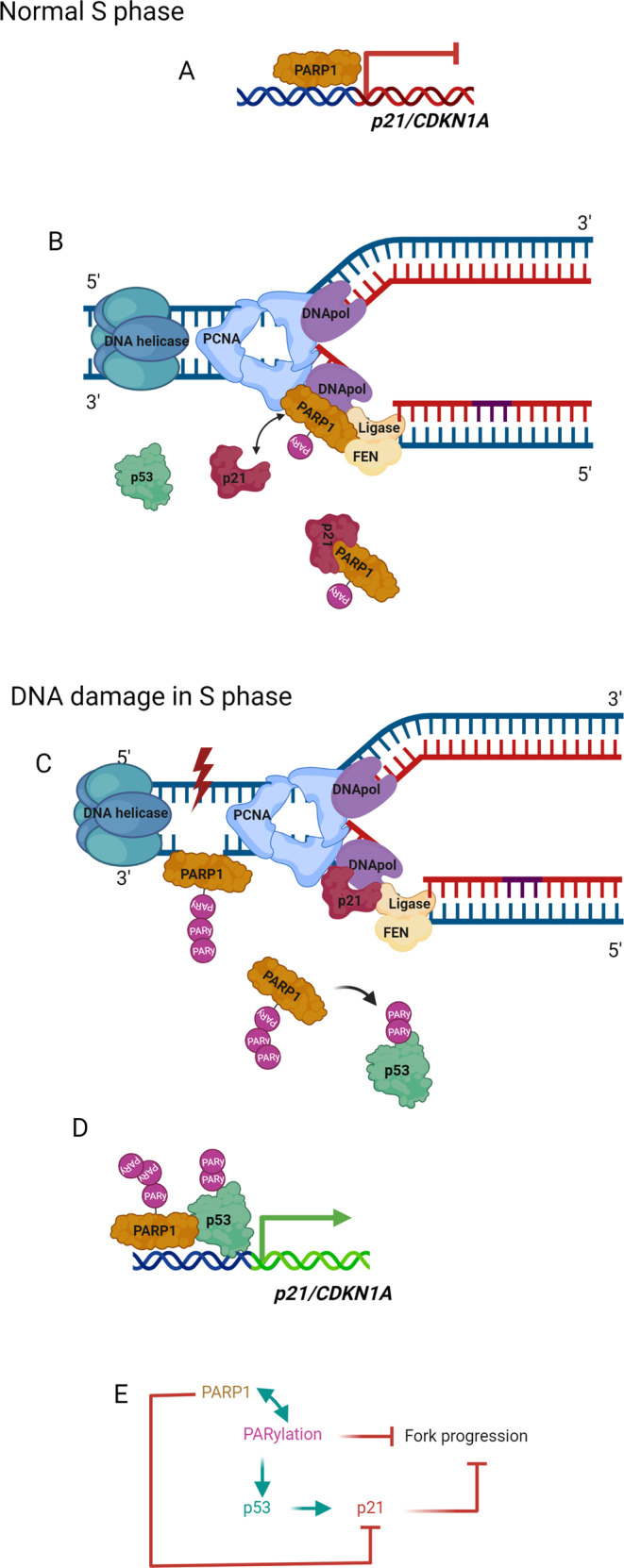
p53 and the Fork Speed Regulatory Network. **A** During unperturbed S phase, inactive PARP1 (Poly(ADP-ribose) Polymerase-1) inhibits transcription of *p21/CDKN1A*. **B** Levels of p53, p21, p21-PARP1 complex, free PARP1 and a low level of PARylation are maintained at a steady state during the normal S phase. **C** Any break in DNA is promptly recognized by PARP1, which triggers its activity. PARylation can promote recruitment of important DDR proteins [83] or can directly inhibit fork progression. **D** When DNA is severely damaged, PARP1 is strongly activated. PARylated PARP1 releases p21 from the p21-PARP1 complexes. PARylated PARP1 is also bound by p53, which helps transactivate p21. p21 blocks the ability of PCNA to activate DNA polymerase δ [87]. Therefore, PARylation and p21 act as suppressors of DNA replication. **E** The overall balance among p53, p21, PARP1, and its activity (PARy) allows maintaining the normal speed of replication fork progression. Together these proteins are part of the Fork Speed Regulatory Network (FSRN) additional components of which await discovery. Green lines indicate activation and red lines negative regulation.

We proposed that any break in front of the replication fork is promptly recognized by PARP1, whose activity is then enhanced (Fig. 2A–D). PARylation can promote the recruitment of key DDR proteins [83] or directly inhibit fork progression. PARylation excess gets enzymatically removed by PARG, allowing the fork to resume its function [84]. Nicks in the leading strand arrest fork progression, whereas nicks in the lagging strand can be bypassed [85] and repaired afterward. When DNA is severely damaged, PARP1 becomes strongly activated and auto-PARylated PARP1 binds p53, transactivating *p21*, while PARylated PARP1 also releases p21 from the p21-PARP1 complexes. After prolonged fork arrest, processive DNA polymerases dissociate from modified PCNA [86] and are replaced by p21. p21 can inhibit PCNA-dependent DNA replication independent of cyclins/CDKs. Furthermore, p21 blocks the ability of PCNA to activate the DNA polymerase δ [87]. Therefore, PARylation and p21 act hand in hand as additive suppressors of DNA replication [52]. Perhaps simultaneously, uncoupled DNA helicases continue to unwind DNA, leaving behind stretches of ssDNA, which are then coated by RPA [88]. RPA signals to ATR, triggering downstream events to activate dormant origins, inhibit the activation of new replication domains, or delay cell cycle progression. During the S phase, cells treated with PARP inhibitors may also accumulate unprocessed Okazaki fragments [89]. Defects in the interplay between p53, PARP1, and p21 lead to supra-threshold acceleration of fork elongation. Altogether, we proposed a concept, in which PARP1, PARylation, and the p53/p21 axis provide a coordinated mechanism, termed the Fork Speed Regulatory Network (FSRN, Fig. 2E), to regulate the speed of fork progression [52]. Future research will undoubtedly identify additional components of this network, and functional interplay among them.

## p53 and the interplay of ribosome biogenesis with rDNA replication

During DNA replication, forks encounter challenges, including damaged bases, non-histone proteins bound to DNA, transcription, repetitive sequences, and DNA in non-canonical structures such as DNA quadruplexes [90]. Ribosomal DNA (rDNA) genes are organized in tandemly repetitive clusters on 5 human acrocentric chromosomes and are vulnerable to recombination. Replication fork barriers (RFBs) at rDNA are necessary to prevent transcription-replication collisions that could lead to fork collapse [91]. Inhibition of rDNA transcription leads to p53-dependent cell cycle arrest, while inhibition of rDNA synthesis, through nucleolar TopBP1 and ATR activation, causes modest p53 elevation [92]. During S phase, p53 prevents DNA topological stress originating from transcription in the nucleolus, thereby promoting normal replication fork progression [55].

Unrestrained oncogenic activity can trigger enhanced nucleotide usage to sustain a high rate of ribosome biogenesis (RiBi) and DNA replication needed to drive cancer cell growth and proliferation, leading to nucleotide pool imbalances and combined replication and nucleolar stress. *De novo* nucleotide synthesis pathways have been increasingly investigated as potential cancer therapy targets. Inhibition of these metabolic pathways often immediately impairs both rRNA synthesis and DNA replication. Both IRBC (impaired ribosome biogenesis checkpoint, which will be discussed more in detail below) and DDR are involved in p53 activation following RiBi defects. ATR activation and imbalanced nucleotide pools were found in cellular and zebrafish models of ribosomal protein (RP) deficiency, and RP loss elicited DDR that likely contributed to p53 activation [93]. The inhibition of the dihydroorotate dehydrogenase (DHODH), an enzyme in the *de novo* pyrimidine synthesis pathway, blocks cancer cell proliferation through induction of replication and nucleolar stress, activation of p53, and the ATR/CHK1 pathway [94]. It is unclear how RS and nucleolar stress are orchestrated in relation to p53, p21, and the cell cycle. In principle, in normal cells, IRBC induces p53-dependent p21-mediated G1 arrest, whereas DDR requires an S-phase entry. Based on experiments using gradual inhibition of the Inosine Monophosphate Dehydrogenase (IMPDH), an enzyme required for *de novo* GMP synthesis, a hierarchical organization was proposed, whereby IRBC provides the primary “nucleotide sensor”, while in a setting of highly effective IMPDH inhibition, p21 degradation takes place and attenuates the IRBC-mediated G1 arrest, allowing entry into the S phase and subsequent DDR activation [95]. These results suggest that IRBC functions to protect cells from genomic instability, explaining some earlier observations regarding the interplay between RS, DDR, and IRBC [95].

Dysregulated rDNA transcription can increase R-loop formation, reflecting conflicts of replication and transcription machineries [96]. Conversely, during erythroid differentiation, inhibition of RNA Pol I evokes transcriptional stress, nucleolar disruption, and activation of the ATR-CHK1-p53 pathway [97]. Therefore, p53 can be activated in at least four ways in response to nucleolar stress, through (i) oncogene-induced RS in the rDNA; (ii) enhanced nucleolar R-loops; (iii) inhibition of rDNA transcription; (iv) impaired RiBi. Biologically, p53 activation can lead to cell cycle delay, cellular senescence, or cell death.

## The ribosome-p53 connection

**Fig. 3:**
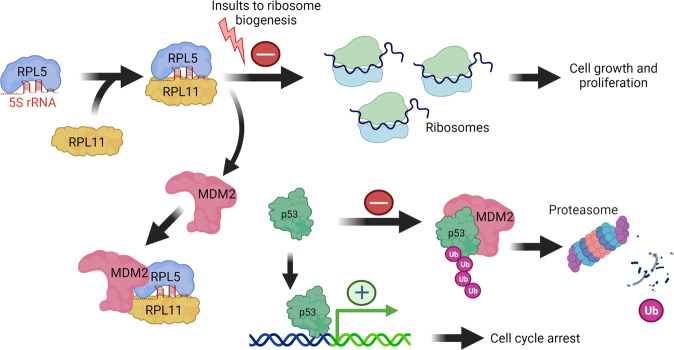
5S RNP complex regulates p53 turnover following insults to ribosome biogenesis. Under normal undisturbed conditions RPL5, RPL11, and 5S rRNA form a pre-ribosomal complex 5S RNP before being incorporated into the nascent large ribosome subunits. Upon ribosome biogenesis stress, such as inhibition of RNA pol I, the surplus 5S RNP complex instead becomes increasingly tethered to MDM2, preventing its inhibitory action towards p53.

Perturbations in RiBi activate a p53-dependent cellular response, and the RiBi machinery is intimately connected to the control of MDM2 and p53. While the hypothesis that nucleolar integrity is linked to p53 turnover [98] and evidence functionally connecting ribosomal protein L11 (RPL11) with MDM2 [99] emerged some 20 years ago, the first clue came already in 1994, namely that MDM2 associates with ribosomal protein L5 (RPL5) and 5S rRNA [100]. Parallel work showed that ribonucleotide synthesis inhibitors triggered a reversible p53-dependent G1 arrest without DNA damage [101]. Furthermore, a dominant-negative form of the RiBi factor BOP1 (Block of Proliferation 1) expressed in fibroblasts, not only blocked RiBi but also triggered a p53-dependent cell-cycle arrest [102]. Such p53-mediated monitoring of nucleolar function and coupling ribosome integrity to the cell cycle inspired the term *nucleolar stress* [102], today also called *ribosomal stress*. A broader hypothesis postulated that the nucleolus senses cellular stress and as soon as nucleolar function is impaired the p53 abundance increases. In parallel, the nucleolar p19Arf (mouse)/p14ARF (human) tumor suppressor was shown to bind and inhibit MDM2 to activate p53 in response to oncogenic signals [103]. Subsequent studies described an essential function of RPL11 in the activation of p53 in cells exposed to low (nanomolar) concentrations of Actinomycin D [99, 104]. Actinomycin D inhibits rRNA synthesis, leading to increased RPL11-RPL5-MDM2 complex formation. RPL11 and RPL5 prevent MDM2-mediated p53 ubiquitination and degradation and this enables p53-mediated transactivation of p53 target genes including p21, inducing cell cycle arrest [105] (Fig. 3). Several other RPs have now been implicated in p53-MDM2 pathway dynamics, including RPL26 that binds the 5’untranslated region of the p53 mRNA boosting translation [106]. In contrast, the RPL5/RPL11/5 S rRNA-mediated checkpoint (see below) operates post-translationally through MDM2 to stabilize p53.

Also other members of the p53 family seem to have a role in ribosome biogenesis. It was shown that RPL11 and RPL5 could associate with the N-terminal domain of p73, and enhance the transcriptional activity of p73 by antagonizing p73-MDM2 interaction [107]. Interestingly, depletion of p73 elicits rRNA processing defects and impaired protein synthesis. Specifically, p73 supports the translation of ribosome biogenesis factors and mitochondrial factors, functions that help to protect cells from oxidative stress [108].

## The impaired ribosome biogenesis checkpoint as a general p53 rheostat

RPL5 and RPL11 binding to MDM2 and p53 activation also requires 5S rRNA [109, 110]. 5S rRNA, RPL5, and RPL11 form the 5S RNP, an assembly intermediate of the large ribosomal subunit [111]. This intermediate particle is considered rather stable and not prone to immediate degradation contrary to other non-ribosome bound RPs. This 5S RNP complex, rather than the individual factors RPL5 and RPL11, binds and regulates MDM2 (Fig. 3). The 5S RNP provides a protected platform where RPL5 and RPL11 reside and escape degradation upon impaired RiBi [110]. Certain mutations in the MDM2 zinc finger disrupt the binding to RPL11/RPL5, preventing p53 stabilization following nucleolar stress [112].

**Fig. 4:**
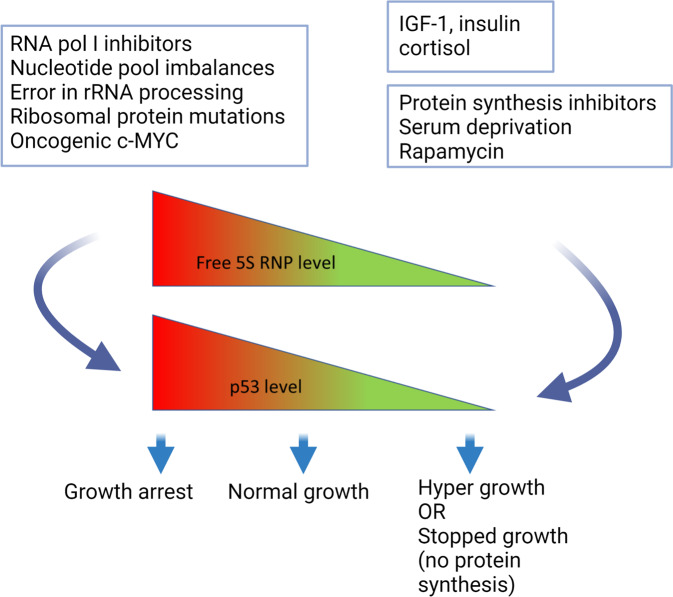
The regulation of 5S RNP and p53 after stress. Levels of free 5S RNP and therefore p53 are influenced by various conditions that affect 5S RNP complex formation and ribosome biogenesis. Conditions that lead to an increase in 5S RNP and p53 are highlighted on the left. In contrast, inhibition of protein synthesis or the opposite scenario under excessive mitogen signaling (IGF-1, insulin) lead to a reduction of free 5S RNP complex and hence p53 (right). The cellular response and dynamics of p53 and 5S RNP are likely to vary over time and among cell types (see main text for more details).

The 5S RNP-MDM2 interaction is enhanced upon alterations in ribosome production that leads to a 5S RNP surplus, and is needed to elevate p53 in response to for example chemotherapeutics (e.g., Actinomycin D, Oxaliplatin, 5-FU), ribonucleotide depletion or loss of ribosomal proteins (other than RPL11, RPL5) [113] (Fig. 4). This mechanism was then termed the Impaired Ribosome Biogenesis Checkpoint (IRBC). With a few exceptions, depleting individual RPs of the large or small ribosomal subunits commonly caused p53-mediated cell cycle delay [114, 115], effects that required the RPL5/RPL11/5S rRNA [116]. Defects in the small subunit also stabilized p53 and this was surprising since large subunit biogenesis occurs independently of the small subunit. It turns out that while depletion of for example RPS6 lowers 40 S production it increases *RPL11* mRNA translation resulting in increased 5S RNP-MDM2 complex formation and p53 activation [117]. Depletion of RiBi factors other than RPs, such as HEATR1 also activates p53 through the IRBC [118]. Yet another example is WDR75, a protein required for pre-rRNA transcription, whose depletion also activates the IRBC/p53 pathway, and interestingly also causes degradation of RPA194 (POLR1A), the catalytic subunit of the RNA pol I complex [115].

Furthermore, oncogenes including E2F-1 and Myc [51], and the ARF tumor suppressor [110] also partially engage the 5S RNP leading to a more robust p53 increase. Taken together, the 5S RNP–MDM2 complex has emerged over the years as a central rheostat to master p53. How is it possible that so many different stressors influence RiBi and p53? A genome-wide RNAi screen focused on 40 S biogenesis showed that several proteins in the small subunit processome, the ubiquitin-proteasome system, and splicing machineries, are critically needed to support proper 40 S biogenesis [119]. Besides, RNA polymerase II activity is also coupled to RiBi through various mechanisms [120, 121]. These findings make it easier to understand how perturbations in diverse cellular functions may affect RiBi leading to activation of the IRBC. A key player besides 5S RNP is the mTOR pathway, and changes in mTOR activity may rapidly converge upon the p53 pathway for example through modulating translation of RP mRNA or by post-translational modifications of regulatory proteins in the MDM2-p53 network [122]. Indeed, several small-molecule mTOR inhibitors blunt the p53 response to nucleolar stress including p53 levels and p53-mediated G1 arrest [123]. However, p53 activation by compounds that disrupt the MDM2-p53 interaction, for example, nutlin-3 appear independent of RPs: depletion of RPL11 had only a modest reducing impact on p53 and p21 levels in nutlin-3 treated cancer cells exposed to Actinomycin D [123]. In contrast, insulin and cortisol signaling lead to decreased p53 levels due to an immediate demand for new ribosomes, a scenario that uses up free cellular RPL11 and RPL5, thereby allowing MDM2 to maintain p53 turnover [124] (Fig. 4). Thus, the 5S RNP appears to be involved in setting the p53 protein level in several situations upon cellular stress. Models have been proposed for how various MDM2-RP interactions or the ribosome itself can regulate p53 or p53-MDM2. Yet, it is not trivial to comprehend the link between p53 and the nucleolar RiBi machinery, and questions remain as to the origin and evolution of this regulatory mechanism.

## Ribosomes, p53 and cancer

The 5S RNP-MDM2-p53 IRBC pathway likely provides a barrier to cancer development, with *RPL5* being frequently altered in human tumors. The IRBC’s anti-cancer role is supported by several findings: (I) RPL5 or RPL11 deficiency impairs p53 activation and may contribute to the increased risk of Diamond Blackfan Anemia (DBA) patients to develop cancer [125]; (II) Mice heterozygous for *Rpl11* are prone to radiation-induced lymphomagenesis, and loss of Rpl11 attenuates p53 activation in response to DNA damage in fibroblasts [126]; (III) Knock-in mice harboring an Mdm2 zinc finger mutation displayed increased Myc-induced lymphomagenesis [127]; (IV) RPL5 mutations occurring in human cancer cell lines blunt p53 activation, indicating 5S RNP haploinsufficiency promotes malignant transformation [128]; (V) *RPL5* heterozygous mutations or deletions occur in up to 34% of breast cancer, melanoma (28%), glioblastoma (11%), and multiple myeloma (up to 30%) [129]. Furthermore, patients with low expression of *RPL5* displayed worse overall survival in glioblastoma and multiple myeloma [130]. RPL5 mutants may impair RiBi, thereby affecting ribosome function and cell growth, while also preventing IRBC by disrupting the RPL5-5S rRNA interaction [128]. Hemizygous deletions of ribosomal protein-encoding genes occurred in 43% of 10,744 cancer samples and cancer cell lines investigated, being underrepresented in *TP53*-intact tumors [131]. Myc-driven B-cell lymphomas are addicted to high-level RiBi and provide a model to assess RiBi-interfering compounds for therapeutic purposes. Loss of RP-MDM2 interaction through mutations in the MDM2 zinc finger accelerated Emu-Myc-induced lymphomagenesis [127]. Consistently, Myc induction leads to increased RiBi and p53 stabilization, in part through increased 5S RNP-MDM2 complex formation [132], while reducing Myc impairs RiBi and decreases 5S RNP and p53. What happens if RPL11 or RPL5 are modulated versus other RPs, since RPL11 and RPL5 are also essential for RiBi? Whereas loss of RPL11 reduced RiBi and cell proliferation similar to depletion of another RP, RPL7a, as expected, only RPL7a depletion and not RPL11 triggered p53-mediated apoptosis through degradation of the anti-apoptotic MCL-1 [133]. These studies illustrate how p53 is tightly woven together with the Myc-driven RiBi program in part through 5S RNP.

## Ribosome biogenesis and p53 in developmental syndromes

Increased p53 activity contributes to pathogenesis of ribosomopathies. In animal models of the human syndromes DBA and Treacher Collins syndrome (TCS) p53 mediates some but not all phenotypes as shown by co-deletion of *Tp53*, or pharmacological inhibition of p53 [134]. In DBA, RP haploinsufficiency results in impaired RiBi, affecting either the small or large subunit, with subsequent activation of p53 and impaired cell growth. It was considered that p53 activation and reprogrammed mRNA translation were independent events in DBA. However, DBA-mimicking *Rps6* haploinsufficiency caused various limb phenotypes attributable to changes in mRNA translation patterns. Surprisingly most of the differential translational changes were restored upon loss of p53 [135]. Thus, p53 activation upon dysfunctional RiBi also involves altered translational control through p53 and its downstream effectors.

**Fig. 5:**
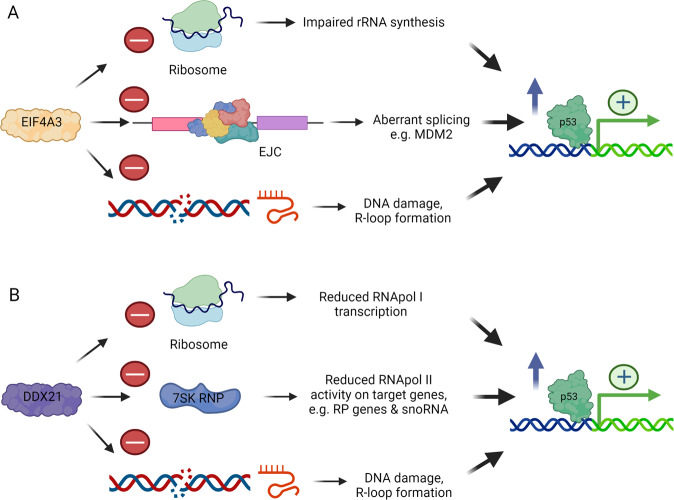
Inhibition of key RNA helicases affects multiple cellular processes triggering p53. **A**. EIF4A3 has essential functional roles in the exon junction complex (EJC) and in ribosome biogenesis. Upon inhibition of EIF4A3 ribosome biogenesis is impaired, certain mRNA including *MDM2* undergo alternative splicing and there is accumulation of R loops and DNA damage, insults converging on p53. **B** DDX21 supports RNA Pol I transcription to boost ribosome biogenesis. It also binds the 7SK RNP complex to aid RNA Pol II-mediated transcription of ribosomal protein (RP) genes and snoRNA. Upon inhibition of DDX21 ribosome biogenesis is impaired, R loops and DNA damage appear and p53 becomes activated.

DDX21 and EIF4A3 are RNA helicases whose loss triggers multifaceted cellular stress responses converging on p53. The RNA binding exon junction complex (EJC) consists of, among others, the proteins MAGOH, RBM8, and EIF4A3. Deficiency in individual EJC components causes abnormal neural development, exemplified by the Richieri Costa Pereira syndrome (RCPS), an autosomal-recessive acrofacial dysostosis presenting with craniofacial malformation and limb defects due to deregulation of EIF4A3 [136]. *Eif4a3* haploinsufficiency in mice triggers microcephaly and aberrant neurogenesis through p53 activation [137]. We have recently shown that EIF4A3 regulates rRNA processing and helps to mitigate nucleolar R-loop formation [138]. While loss of other EJC proteins also induces p53, EIF4A3 appears more intimately connected to RiBi through its partial nucleolar localization, RNA binding, and helicase activity. EIF4A3 depletion also triggered aberrant splicing patterns of *MDM2* and DNA damage, while inactivation of 5S RNP only partially attenuated the p53 response in EIF4A3-depleted cells [138]. Interestingly, alternative splicing and *MDM2* exon 3 skipping was described in mice deficient in the pre-mRNA splicing factor Eftud2, resulting in p53 activation [139]. Taken together, targeting of EIF4A3 resulted in altered *MDM2* splicing, impaired RiBi, activation of IRBC, DNA damage, as well as p53 elevation and apoptosis [138] (Fig. 5A). As *MDM2* is altered by splicing, yet there is nucleolar stress, DNA damage and IRBC activation, p53 induction cannot in such a complex scenario be solely attributed to only one pathway or mechanism in RCPS. Nevertheless, while EIF4A3 is an essential protein these combined effects, including mis-splicing of *MDM2*, should be further explored in cancer treatment.

DDX21 (previously known as RH-II/Gu alpha), is a DEAD-box RNA helicase also involved in RiBi and nucleolar function. DDX21 associates with genes actively transcribed by RNA Pol I and II and unwinds R-loops. In the nucleolus, DDX21 occupies transcribed rDNA genes and promotes rRNA synthesis. Depletion of DDX21 activates the IRBC and p53, but also leads to accumulation of R-loops and DNA damage [140, 141] (Fig. 5B). TCS, the craniofacial disorder caused by defective RNA Pol I, particularly its cofactor TCOF1, features enhanced p53 activity in neural crest cells and apoptosis [121]. In TCS, DDX21 re-localizes from the nucleolus to the nucleoplasm, and is lost from its chromatin targets, with ensuing nucleolar stress and DDR activation. Surprisingly, preventing DDX21 loss from the nucleolus and chromatin rescues apoptosis and craniofacial phenotypes in TCS, thus revealing an unexpected function of DDX21 in the nucleolar stress response and cell fate determination. Interestingly, treatment of cancer cells with PARPi reduced DDX21 nucleolar localization, downregulated RiBi and impaired cell growth [142]. Together, the studies on EIF4A3 and DDX21 exemplify involvement of helicases in cellular functions related to RiBi, and how closely related they are to the p53 pathway. This work also reveals opportunities for therapeutic intervention in both cancer and developmental syndromes like TCS or RCPS, not least by interference with p53.

## Targeting RiBi in cancer, new insights related to p53

As p53 responds to diverse stress signals, compounds modulating such converging pathways, including replication stress tolerance as a targetable cancer vulnerability [143–145], may complement or potentiate agents inducing RiBi stress. It appears that RiBi inhibition in cells derived from solid tumors commonly triggers cytostatic effects rather than apoptosis. One possible explanation is that inhibition of RNA Pol I predominantly induces cell cycle arrest accompanied by autophagy, the latter possibly allowing cancer cells to escape cell death. Indeed, blocking autophagy sensitized cancer cells to RNA Pol I inhibition [146]. In this setting, the FDA-approved anti-malaria drug amodiaquine might be repositioned for cancer therapy. We found that amodiaquine triggers degradation of the RNA Pol I catalytic subunit RPA194, in a manner independent of the known amodiaquine-induced autophagic-lysosomal blockade [147]. Furthermore, impaired RiBi is also seen in response to a range of cellular stressors, including nutrient deprivation, altered redox balance, DNA damage, or hypoxia [148]. Notably, impairment of almost any stage in RiBi triggers IRBC leading to p53 activation. This fascinating circumstance aligns well with p53 activation being regarded as an important goal of cancer chemotherapy. Blocking RiBi is such a strong p53-activating signal. Several RNA Pol I inhibitors have been investigated including BMH-21 and CX-5461 [149]. However, many such drugs are not genuinely specific for RNA Pol I. Being a DNA intercalator (BMH-21), or a TOP2 inhibitor (CX-5461) such compounds may interfere with other DNA related processes, beyond the nucleolar rDNA. Yet it is striking that many standard-of-care chemotherapeutics, including 5-FU, oxaliplatin, actinomycin D, and doxorubicin have a robust RiBi-inhibitory activity [150]. Conceptually the approach to target RiBi would largely rely on a functioning wt p53 pathway. However, in most tumors, p53 is inactivated by mutations or other means, such as through reduced levels of the 5S RNP component RPL5 or MDM2 overexpression. Despite this, there are reasons for cautious optimism. Prolonged blockade of RiBi in p53-defective cells leads to a halted cell growth through various p53-independent mechanisms sensing and signaling nucleolar stress [151]. Additionally, other therapeutic strategies restore the function of mutant p53, or re-introduce WT-p53 [152].

## Concluding remarks

Among the fundamental biological processes mechanistically linked to p53, the fields of ribosome biogenesis and replication stresses, including their involvement in human pathologies including cancer, have witnessed striking progress in recent years. In this review, we provide concise background information about the roles of p53 in DNA replication and ribosome biogenesis, with emphasis on deregulation of these mechanisms and the roles of p53 pathways in the maintenance of cell homeostasis, including the emerging functional links between such stress response mechanisms. Apart from providing an overview of current mechanistic understanding of these rapidly evolving areas of biomedicine, we also briefly outline the vulnerability of cancer cells due to their adaptation to chronic ribosome biogenesis stress, with examples of emerging compounds to target such aberrant conditions therapeutically. Finally, throughout the article, we also point out some burning open questions the elucidation of which requires further research, both basic and clinical.
